# Announcement: Third European Symposium on Calcium Binding Proteins in Normal and Transformed Cells

**DOI:** 10.1155/MBD.1994.79

**Published:** 1994


### THIRD EUROPEAN SYMPOSIUM ON CALCIUM BINDING PROTEINS IN NORMAL AND TRANSFORMED CELLS

### Zurich, March 6-9, 1994

**Symposium Secretariat**
Ms. M. Killen
University Children's Hospital, Div. of Clinical Chemistry, Steinwiesstr. 75, CH-8032 Zurich, Switzerland, TEL +41 I 266 7542, FAX +41 1 266 7171

**Bank**
Swiss Bank Corporation, CH-8030 Zurich-Hottingen, Acct.no. R3-570'009.0 320

**Organizing Committee**
R. Donato (Italy), J. Haiech (France), C. Heizmann (Switzerland), R. Pochet (Belgium)

**Local Committee**
C. Heizmann, M. Killen, B. Schafer

**Plenary Lectures**
**M. Berridge** (Cambridge): The spatiotemporal aspects of calcium signalling
**R. Huber** (Munich): Annexins, structure, calcium binding and ion channel properties
**R.Y. Tsien** (La Jolla): New control mechanisms and visualization techniques for cytosolic calcium

**Speakers and Chairpersons**(provisional)
J. Baudier (Grenoble), M. Berchtold (Zurich), M. Celio (Fribourg), S. Christakos (Newark), J. Cox (Geneva), R. Donato (Perugia), M. Doree (Montpellier), C.S. Fullmer (Ithaca), V. Gerke (Göttingen), J. Haiech (Marseille), C. Heizmann (Zurich), H. Hidaka (Nagoya), C. Klee (Bethesda), R.H. Kretsinger (Charlottesville), J. Meldolesi (Milano), T. Meyer (Durham), S.E. Moss (London), R. Pochet (Bruxelles), B. Schafer (Zurich), E. Strehler (Rochester), T. Tanaka (Tsu), L. Van Eldik (Nashville), R.H. Wasserman (Ithaca), M. Watterson (Nashville), R.J.P. Williams (Oxford)

**Scientific Program**
The scientific sessions will include the following topics:
CALCIUM SIGNALLING, EF-HAND $Ca^{2+}$ -BINDING PROTEINS AND ANNEXINS: protein and gene structures, chromosomal assignments, regulation of gene expression, tissue localization, metal binding properties, posttranslational modifications, target proteins, novel $Ca^{2+}$-binding proteins, intra- and extracellular functions, e.g., roles in cell proliferation and apoptosis, differentiation, nuclear functions.
CALCIUM-BINDING PROTEINS IN PATHOLOGY: involvement of $Ca^{2+}$-binding proteins in neurodegenerative disorders, seizures, tumor progression, cardiovascular diseases, immunosuppression and inflammation
CALCIUM-BINDING PROTEINS IN PHARMACOLOGY AND DIAGNOSTICS: drug binding, cell- and tissue-specific markers, classification of tumors, elevations in body fluids in various diseased states.

**General Information**

VENUE: The conference will take place at the Federal Institute of Technology (ETH), Auditorium Maximum (Main Lecture Hall), Zurich, Switzerland

LANGUAGE: English is the official language of the conference.

REGISTRATION: The number of participants is limited. The registration fee includes access to all scientific sessions, a miniposter and abstracts book, welcome reception, coffee breaks, and banquet.

| | before Nov. 1,1993 | after Nov. 1, 1993 |
|---|---|---|
| registration fee | SFr. 300.-- | SFr. 400.-- |
| students | SFr. 250.-- | SFr. 400.-- |

HOTEL ACCOMMODATIONS: Hotel Zurich, Neumühlequai 42, CH-8001 Zurich, is the Conference Hotel. The hotel is in the center of the city within walking distance of the lecture hall and the train station. The hotel offers special rates (approx. 50% reduced) for all participants, including one night before and after the meeting. We recommend that young participants take the opportunity to share the very large and comfortable rooms at Hotel Zurich where all the speakers will stay. Hotel expenses are to be paid directly to the hotel. The hotel staff will also help you with organizing sightseeing trips in Zurich, in the surrounding areas, to ski resorts etc.

LUNCHES: Lunch is available at the ETH Cafeteria for SFr. 9.50.

TRANSPORTATION: SWISSAIR has been appointed Official Carrier to the symposium.

Airport-City hotel bus available from Terminal B, arrival level.

The Federal Institute of Technology is within walking distance of the hotel and the main train station. Parking is limited.

WEATHER IN ZURICH IN MARCH: average temperature 4.7°C, average precipitation 60mm.

SOCIAL PROGRAM

Sunday, March 6,1994: *Registration:* Hotel Zurich, Neumühlequai 42, 8001 Zurich, 13.00 - 20.00 h

*Welcome Reception:* Hotel Zurich, Neumühlequai 42, 8001 Zurich, 19.00 - 23.00 h

Tuesday, March 8,1994: *Banquet:* University of Zurich, Lichthof, Rämistr. 71, 8001 Zurich, 19.00-23.00 hr.

*Excursions*: Contact Hotel Zurich

**MINIPOSTERS**: A feature of this symposium will be the miniposter book from which selected communications will be presented at Round Tables each day of the meeting. It is intended to stimulate as much as possible discussion among the young participants.

In order to reach this goal you are invited to submit a 'miniposter' which will be bound in a book and sent to you before the meeting.

The miniposters (inclusive of text, tables and figures) should be prepared on sheets within a frame of 25x38 cm (97/8x15"). Left margin 2.5 cm. These will be reduced to their final size of 17x26 cm (63/4x101/4"). Please use a "lettertype" printer - most matrix printers are not suitable. Electron micrographs may be included.

SLIDE AND FILM PROJECTION: Facilities are available for 24 x 36 mm slides, simultaneous projection of two slides; overhead projection; VHS videos; 8 mm and 16 mm films.

### IMPORTANT DEADLINES

**November 1, 1993:** Deadline for registration at reduced rate. Deadline for submission of miniposters.

**January 31, 1994:** Deadline for hotel reservations.

